# Poly[bis[3,10-bis(2-hydroxyethyl)-1,3,5,8,10,12-hexa­azacyclo­tetra­deca­ne]tetra-μ-cyanido-tetracyanidodicopper(II)molybdenum(IV)] tetra­hydrate]

**DOI:** 10.1107/S1600536808036131

**Published:** 2008-11-13

**Authors:** Hu Zhou, Ying-Ying Chen, Wen-Yan Liu, Ai-Hua Yuan

**Affiliations:** a School of Materials Science and Engineering, Jiangsu University of Science and Technology, Zhenjiang 212003, People’s Republic of China

## Abstract

In the title complex, {[Cu_2_Mo(CN)_8_(C_12_H_30_N_6_O_2_)_2_]·4H_2_O}_*n*_, the polyhedron around Mo has site symmetry 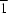 with a distorted square-antiprismatic shape, while the Cu atom (2 symmetry) is in a distorted axially elongated octa­hedral coordination environment. The uncoordinated water molecule is disordered over three sites with occupancies of 0.445 (7), 0.340 (7) and 0.215 (7). Mo and Cu atoms acting as basic components are connected by an Mo—CN—Cu—NC—Mo— linkage to form a distorted diamond-like network. Additional hydrogen bonding between the N—H groups and the water molecules stabilizes this arrangement.

## Related literature

For background information, see: Larionova *et al.* (2004 ▶); Przychodzeń *et al.* (2006 ▶). For related structures, see: Chen *et al.* (2007 ▶); Zhou *et al.* (2007 ▶; 2008 ▶). For literature related to the synthesis, see: Suh & Kang (1988 ▶); Leipoldt *et al.* (1974 ▶).
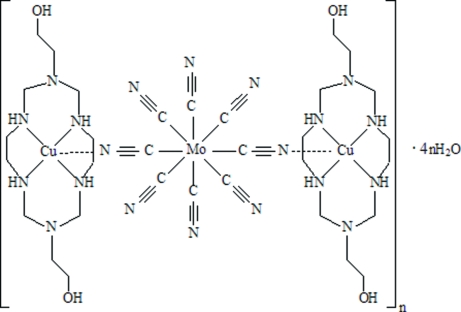


## Experimental

### 

#### Crystal data

[Cu_2_Mo(CN)_8_(C_12_H_30_N_6_O_2_)_2_]·4H_2_O
*M* *_r_* = 1084.08
Tetragonal, 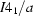

*a* = 20.0707 (10) Å
*c* = 15.4380 (17) Å
*V* = 6218.9 (8) Å^3^
*Z* = 4
Mo *K*α radiation
μ = 0.93 mm^−1^
*T* = 293 (2) K
0.32 × 0.28 × 0.26 mm

#### Data collection

Bruker SMART APEXII diffractometer
Absorption correction: multi-scan (*SADABS*; Bruker, 2002 ▶) *T*_min_ = 0.75, *T*_max_ = 0.79
16289 measured reflections
3053 independent reflections
2459 reflections with *I* > 2σ(*I*)
*R*_int_ = 0.058

#### Refinement

*R*[*F* ^2^ > 2σ(*F* ^2^)] = 0.043
*wR*(*F* ^2^) = 0.103
*S* = 1.06
3053 reflections
165 parameters
1 restraint
H-atom parameters constrained
Δρ_max_ = 0.23 e Å^−3^
Δρ_min_ = −0.38 e Å^−3^

### 

Data collection: *APEX2* (Bruker, 2004 ▶); cell refinement: *SAINT* (Bruker, 2004 ▶); data reduction: *SAINT*; program(s) used to solve structure: *SHELXS97* (Sheldrick, 2008 ▶); program(s) used to refine structure: *SHELXL97* (Sheldrick, 2008 ▶); molecular graphics: *SHELXTL* (Sheldrick, 2008 ▶); software used to prepare material for publication: *SHELXTL*.

## Supplementary Material

Crystal structure: contains datablocks I, New_Global_Publ_Block. DOI: 10.1107/S1600536808036131/pv2114sup1.cif

Structure factors: contains datablocks I. DOI: 10.1107/S1600536808036131/pv2114Isup2.hkl

Additional supplementary materials:  crystallographic information; 3D view; checkCIF report

## Figures and Tables

**Table 1:** Hydrogen-bond geometry (Å, °)

*D*—H⋯*A*	*D*—H	H⋯*A*	*D*⋯*A*	*D*—H⋯*A*
N3—H3*C*⋯N2^i^	0.93	2.44	3.261 (4)	147
N5—H5*C*⋯O2^ii^	0.93	2.38	3.291 (8)	165
O1—H1*C*⋯O4^iii^	0.85	2.48	3.149 (7)	136
O4—H4*D*⋯N2^iv^	0.85	2.11	2.729 (6)	129
O4—H4*E*⋯O2^v^	0.85	2.47	3.279 (9)	159

Symmetry codes: (i) 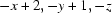; (ii) 
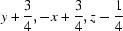
; (iii) 
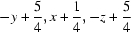
; (iv) 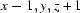; (v) 
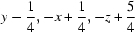
.

**Fig. 1.**
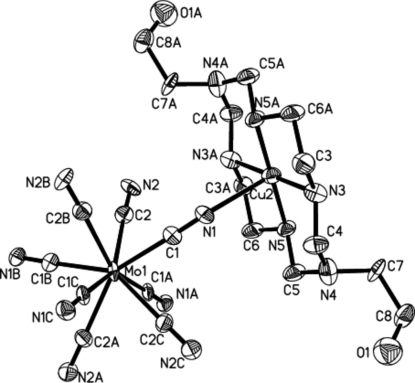
ORTEP drawing of the title complex, showing the atom labeling. Hydrogen atoms and water molecules are omitted for clarity. Symmetry codes: A: 2-x, 1-y, -z; B: 2-x, 0.5-y, z; C: 1.25-y, -0.75+x, 0.25-z.

**Fig. 2.**
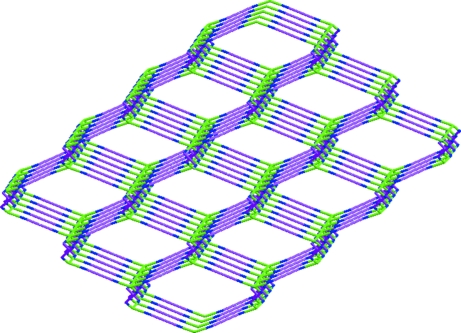
Topological depiction of the title complex, where the Mo nodes and the Cu connectors are shown in pink and green colors, respectively.

## supplementary crystallographic information

### Comment

Recently, octacyanometalates [*M*(CN)_8_]^3-/4-^ (*M* = Mo, W and Nb) have been found to be versatile building blocks and investigated extensively (Larionova *et al.*, 2004; Przychodzeń *et al.*, 2006). For [CuL]^2+^/[*M*(CN)_8_]^3-/4-^ (*L* = macrocyclic ligands) bimetallic systems, macrocyclic ligands block partly coordination sites of the metal ions and release their apical ones, which can be used to construct cyano-bridged bimetallic complexes with octacyanometalate [*M*(CN)_8_]^3-/4-^ ions. In a recent study, we chose [CuL]^2+^ (*L* = 3,10-diethanol-1,3,5,8,10,12-hexaazacyclotetradecane) as building block to synthesize successfully an octacyanometalate-based bimetallic complex [CuL]_2_[Mo(CN)_8_].4H_2_O, (I), with a distorted diamond network.

The title complex crystallizes in the tetragonal (*I*4_1_*/a*) space group. As displayed in Fig. 1, the Mo atom is coordinated by eight CN groups in a distorted square antiprism. The Cu atom is in a distorted axially elongated octahedral coordination environment, in which four nitrogen atoms from the ligand (*L*) occupy the equatorial positions, while the axial sites are occupied by two nitrogen atoms from the bridging cyanide groups on different [Mo(CN)_8_]^4-^ anions. The bonding parameters of the macrocyclic ligand *L* are reminiscent of those found in related complexes reported previously (Chen *et al.*, 2007).

As shown in Fig. 2, Mo and Cu atoms acting as basic components (linker and connector, respectively) are connected by the Mo—CN—Cu—NC—Mo— linkages to form a three-dimensional structure. The network is composed of [CuL]^2+^ unit that is linked *via* cyanides to adjacent four-connected [Mo(CN)_8_(*µ*-CN)_4_]^4-^ centers. From a topological standpoint, each [Mo(CN)_8_] unit is a tetrahedral four-connecting node. These nodes are linked to four adjacent [Mo(CN)_8_] units by the [CuL] units, acting as linear two-connectors. The result is a distorted diamond network (Fig. 2), which is similar to octacyanometalate-based bimetallic complexes reported previously (Zhou *et al.*, 2007; 2008).

### Experimental

Well shaped brown crystals of the title complex suitable for X-ray single-crystal structure determination were grown at room temperature by slow diffusion of a DMF solution (15 ml) of [CuL](ClO_4_)_2_ (0.30 mmol) (Suh and Kang, 1988) and an aqueous solution (15 ml) of K_4_[Mo(CN)_8_].2H_2_O (0.15 mmol) (Leipoldt *et al.*, 1974) in a U-shaped tube containing agar for about one month. The resulting crystals were collected, washed with H_2_O and dried in air.

### Refinement

All hydrogen atoms except for hydrogen atoms bound to water molecules were calculated at idealized positions with C–H = 0.99, N–H = 0.93 and O–H = 0.85 Å and included in the refinement in a riding mode with U_iso_ for H assigned as 1.2 times U_eq_ of the attached atoms. The O atom of the water of hydration molecule was disordered over three sites with unequal site occupancy factors at locations O2, O3 and O4. The H atoms of the disordered water molecule were located from difference maps and were included in the refinements at geometrically idealized positions with O–H distances 0.85 Å and U_iso_ assigned as 1.2 times U_eq_ of the attached O atoms.

### Crystal data

**Table:**

[Cu_2_Mo(CN)_8_(C_12_H_30_N_6_O_2_)_2_]·4H_2_O	*Z* = 4
*M**_r_* = 1084.08	*F*_000_ = 2256
Tetragonal, *I*4_1_/*a*	*D*_x_ = 1.158 Mg m^−^^3^
Hall symbol: -I 4ad	Mo *K*α radiation λ = 0.71073 Å
*a* = 20.0707 (10) Å	Cell parameters from 3093 reflections
*b* = 20.0707 (10) Å	θ = 2.6–21.2º
*c* = 15.4380 (17) Å	µ = 0.93 mm^−^^1^
α = 90º	*T* = 293 (2) K
β = 90º	Block, brown
γ = 90º	0.32 × 0.28 × 0.26 mm
*V* = 6218.9 (8) Å^3^	

### Data collection

**Table:**

Bruker SMART APEXII diffractometer	3053 independent reflections
Radiation source: sealed tube	2459 reflections with *I* > 2σ(*I*)
Monochromator: graphite	*R*_int_ = 0.058
*T* = 293(2) K	θ_max_ = 26.0º
phi and ω scans	θ_min_ = 1.7º
Absorption correction: multi-scan(SADABS; Bruker, 2002)	*h* = −24→24
*T*_min_ = 0.75, *T*_max_ = 0.79	*k* = −19→24
16289 measured reflections	*l* = −16→19

### Refinement

**Table:**

Refinement on *F*^2^	Secondary atom site location: difference Fourier map
Least-squares matrix: full	Hydrogen site location: inferred from neighbouring sites
*R*[*F*^2^ > 2σ(*F*^2^)] = 0.043	H-atom parameters constrained
*wR*(*F*^2^) = 0.103	*w* = 1/[σ^2^(*F*_o_^2^) + (0.0515*P*)^2^ + 3.1936*P*] where *P* = (*F*_o_^2^ + 2*F*_c_^2^)/3
*S* = 1.06	(Δ/σ)_max_ < 0.001
3053 reflections	Δρ_max_ = 0.23 e Å^−^^3^
165 parameters	Δρ_min_ = −0.38 e Å^−^^3^
1 restraint	Extinction correction: none
Primary atom site location: structure-invariant direct methods	

### Special details

**Table:**

Geometry. All esds (except the esd in the dihedral angle between two l.s. planes) are estimated using the full covariance matrix. The cell esds are taken into account individually in the estimation of esds in distances, angles and torsion angles; correlations between esds in cell parameters are only used when they are defined by crystal symmetry. An approximate (isotropic) treatment of cell esds is used for estimating esds involving l.s. planes.
Refinement. Refinement of *F*^2^ against ALL reflections. The weighted *R*-factor *wR* and goodness of fit *S* are based on *F*^2^, conventional *R*-factors *R* are based on *F*, with *F* set to zero for negative *F*^2^. The threshold expression of *F*^2^ > σ(*F*^2^) is used only for calculating *R*-factors(gt) *etc*. and is not relevant to the choice of reflections for refinement. *R*-factors based on *F*^2^ are statistically about twice as large as those based on *F*, and *R*- factors based on ALL data will be even larger.

### Fractional atomic coordinates and isotropic or equivalent isotropic displacement parameters (Å^2^)

**Table:**

	*x*	*y*	*z*	*U*_iso_*/*U*_eq_	Occ. (<1)
C1	0.96688 (13)	0.34611 (15)	0.08263 (19)	0.0392 (6)	
C2	1.06251 (16)	0.27104 (17)	0.0121 (2)	0.0485 (7)	
C3	0.86789 (19)	0.5451 (2)	0.0135 (2)	0.0615 (9)	
H3A	0.8364	0.5817	0.0269	0.074*	
H3B	0.8476	0.5025	0.0322	0.074*	
C4	0.92199 (19)	0.5426 (2)	0.1513 (2)	0.0610 (9)	
H4A	0.8863	0.5716	0.1746	0.073*	
H4B	0.9081	0.4957	0.1597	0.073*	
C5	1.0360 (2)	0.5073 (2)	0.1850 (2)	0.0627 (10)	
H5A	1.0195	0.4618	0.1971	0.075*	
H5B	1.0715	0.5172	0.2277	0.075*	
C6	1.1181 (2)	0.4567 (2)	0.0813 (2)	0.0649 (10)	
H6A	1.1023	0.4126	0.1013	0.078*	
H6B	1.1591	0.4682	0.1137	0.078*	
C7	0.99694 (18)	0.62268 (18)	0.2106 (2)	0.0570 (9)	
H7A	0.9623	0.6491	0.1805	0.068*	
H7B	1.0397	0.6317	0.1808	0.068*	
C8	1.0028 (2)	0.6495 (2)	0.3018 (3)	0.0694 (11)	
H8A	1.0505	0.6519	0.3175	0.083*	
H8B	0.9848	0.6954	0.3030	0.083*	
Cu2	1.0000	0.5000	0.0000	0.04929 (18)	
Mo1	1.0000	0.2500	0.1250	0.03165 (14)	
N1	0.94948 (13)	0.39753 (13)	0.05790 (17)	0.0467 (6)	
N2	1.09533 (14)	0.28414 (15)	−0.04505 (19)	0.0554 (7)	
N3	0.92993 (13)	0.55566 (15)	0.05812 (19)	0.0539 (7)	
H3C	0.9417	0.6002	0.0514	0.065*	
N4	0.98158 (17)	0.55421 (16)	0.1980 (3)	0.0737 (9)	
N5	1.06603 (14)	0.50813 (15)	0.09613 (16)	0.0500 (7)	
H5C	1.0866	0.5493	0.0896	0.060*	
O1	0.9722 (2)	0.6148 (2)	0.3594 (3)	0.1107 (12)	
H1C	0.9917	0.5774	0.3651	0.133*	
O2	0.1088 (4)	0.4119 (4)	0.3446 (5)	0.072 (3)	0.340 (7)
H2A	0.0857	0.4473	0.3454	0.087*	0.340 (7)
H2B	0.0994	0.3893	0.3896	0.087*	0.340 (7)
O3	0.2794 (8)	0.5343 (7)	0.0656 (10)	0.088 (6)	0.215 (7)
H3D	0.2957	0.5708	0.0836	0.105*	0.215 (7)
H3E	0.3107	0.5076	0.0526	0.105*	0.215 (7)
O4	0.2103 (3)	0.2935 (3)	0.8613 (3)	0.065 (2)	0.445 (7)
H4D	0.1814	0.3166	0.8882	0.078*	0.445 (7)
H4E	0.1970	0.2534	0.8577	0.078*	0.445 (7)

### Atomic displacement parameters (Å^2^)

**Table:**

	*U*^11^	*U*^22^	*U*^33^	*U*^12^	*U*^13^	*U*^23^
C1	0.0211 (12)	0.0461 (17)	0.0503 (17)	0.0025 (11)	−0.0005 (11)	0.0068 (13)
C2	0.0437 (17)	0.0534 (19)	0.0485 (18)	−0.0006 (14)	0.0066 (14)	−0.0020 (15)
C3	0.058 (2)	0.064 (2)	0.063 (2)	−0.0125 (18)	−0.0127 (17)	0.0055 (18)
C4	0.058 (2)	0.083 (3)	0.0428 (19)	−0.0050 (19)	0.0110 (15)	0.0042 (17)
C5	0.068 (2)	0.079 (3)	0.0419 (18)	0.0000 (19)	−0.0184 (17)	−0.0064 (17)
C6	0.072 (2)	0.079 (3)	0.044 (2)	−0.007 (2)	−0.0283 (18)	0.0000 (17)
C7	0.060 (2)	0.056 (2)	0.0550 (19)	−0.0147 (16)	0.0152 (16)	−0.0383 (16)
C8	0.071 (2)	0.068 (2)	0.069 (3)	−0.003 (2)	0.019 (2)	−0.024 (2)
Cu2	0.0514 (3)	0.0560 (3)	0.0405 (3)	−0.0020 (3)	−0.0059 (2)	0.0008 (2)
Mo1	0.01805 (15)	0.01805 (15)	0.0589 (3)	0.000	0.000	0.000
N1	0.0459 (14)	0.0452 (14)	0.0490 (15)	0.0031 (11)	−0.0036 (12)	0.0120 (12)
N2	0.0549 (17)	0.0586 (17)	0.0528 (17)	−0.0083 (14)	0.0019 (14)	0.0287 (14)
N3	0.0405 (14)	0.0671 (18)	0.0540 (16)	0.0017 (13)	0.0018 (12)	0.0123 (14)
N4	0.071 (2)	0.0478 (18)	0.103 (3)	−0.0030 (15)	0.018 (2)	−0.0012 (17)
N5	0.0521 (16)	0.0572 (17)	0.0406 (14)	0.0061 (13)	−0.0163 (12)	−0.0079 (12)
O1	0.115 (3)	0.113 (3)	0.104 (3)	0.004 (2)	0.011 (2)	0.006 (2)
O2	0.060 (5)	0.092 (6)	0.065 (5)	0.023 (4)	−0.024 (4)	−0.028 (4)
O3	0.097 (11)	0.063 (8)	0.103 (11)	−0.006 (7)	0.041 (9)	−0.006 (8)
O4	0.064 (4)	0.091 (5)	0.041 (3)	−0.014 (3)	0.016 (2)	0.009 (3)

### Geometric parameters (Å, °)

**Table:**

C1—N1	1.154 (4)	C8—O1	1.285 (5)
C1—Mo1	2.143 (3)	C8—H8A	0.9900
C2—N2	1.132 (4)	C8—H8B	0.9900
C2—Mo1	2.188 (3)	Cu2—N5^i^	1.996 (2)
C3—N3	1.439 (4)	Cu2—N5	1.996 (2)
C3—C6^i^	1.490 (5)	Cu2—N3	2.008 (3)
C3—H3A	0.9900	Cu2—N3^i^	2.008 (3)
C3—H3B	0.9900	Cu2—N1^i^	2.461 (3)
C4—N4	1.416 (5)	Cu2—N1	2.461 (3)
C4—N3	1.470 (5)	Mo1—C1^ii^	2.143 (3)
C4—H4A	0.9900	Mo1—C1^iii^	2.143 (3)
C4—H4B	0.9900	Mo1—C1^iv^	2.143 (3)
C5—N4	1.457 (5)	Mo1—C2^iii^	2.188 (3)
C5—N5	1.499 (5)	Mo1—C2^iv^	2.188 (3)
C5—H5A	0.9900	Mo1—C2^ii^	2.188 (3)
C5—H5B	0.9900	N3—H3C	0.9300
C6—N5	1.487 (5)	N5—H5C	0.9300
C6—C3^i^	1.490 (5)	O1—H1C	0.8501
C6—H6A	0.9900	O2—H2A	0.8500
C6—H6B	0.9900	O2—H2B	0.8500
C7—N4	1.422 (4)	O3—H3D	0.8500
C7—C8	1.512 (5)	O3—H3E	0.8500
C7—H7A	0.9900	O4—H4D	0.8498
C7—H7B	0.9900	O4—H4E	0.8498
			
N1—C1—Mo1	178.4 (3)	N3^i^—Cu2—N1	89.19 (10)
N2—C2—Mo1	177.5 (3)	N1^i^—Cu2—N1	180.00 (11)
N3—C3—C6^i^	108.1 (3)	C1^ii^—Mo1—C1^iii^	95.35 (5)
N3—C3—H3A	110.1	C1^ii^—Mo1—C1	144.45 (16)
C6^i^—C3—H3A	110.1	C1^iii^—Mo1—C1	95.35 (5)
N3—C3—H3B	110.1	C1^ii^—Mo1—C1^iv^	95.35 (5)
C6^i^—C3—H3B	110.1	C1^iii^—Mo1—C1^iv^	144.45 (16)
H3A—C3—H3B	108.4	C1—Mo1—C1^iv^	95.35 (5)
N4—C4—N3	112.2 (3)	C1^ii^—Mo1—C2^iii^	145.00 (12)
N4—C4—H4A	109.2	C1^iii^—Mo1—C2^iii^	76.18 (12)
N3—C4—H4A	109.2	C1—Mo1—C2^iii^	70.56 (12)
N4—C4—H4B	109.2	C1^iv^—Mo1—C2^iii^	75.69 (12)
N3—C4—H4B	109.2	C1^ii^—Mo1—C2^iv^	70.56 (12)
H4A—C4—H4B	107.9	C1^iii^—Mo1—C2^iv^	75.69 (12)
N4—C5—N5	114.8 (3)	C1—Mo1—C2^iv^	144.99 (12)
N4—C5—H5A	108.6	C1^iv^—Mo1—C2^iv^	76.18 (12)
N5—C5—H5A	108.6	C2^iii^—Mo1—C2^iv^	74.44 (17)
N4—C5—H5B	108.6	C1^ii^—Mo1—C2	75.69 (12)
N5—C5—H5B	108.6	C1^iii^—Mo1—C2	144.99 (12)
H5A—C5—H5B	107.5	C1—Mo1—C2	76.17 (12)
N5—C6—C3^i^	107.5 (3)	C1^iv^—Mo1—C2	70.55 (12)
N5—C6—H6A	110.2	C2^iii^—Mo1—C2	129.35 (11)
C3^i^—C6—H6A	110.2	C2^iv^—Mo1—C2	129.35 (11)
N5—C6—H6B	110.2	C1^ii^—Mo1—C2^ii^	76.17 (12)
C3^i^—C6—H6B	110.2	C1^iii^—Mo1—C2^ii^	70.55 (12)
H6A—C6—H6B	108.5	C1—Mo1—C2^ii^	75.69 (12)
N4—C7—C8	119.2 (4)	C1^iv^—Mo1—C2^ii^	144.99 (12)
N4—C7—H7A	107.5	C2^iii^—Mo1—C2^ii^	129.36 (11)
C8—C7—H7A	107.5	C2^iv^—Mo1—C2^ii^	129.35 (11)
N4—C7—H7B	107.5	C2—Mo1—C2^ii^	74.44 (17)
C8—C7—H7B	107.5	C1—N1—Cu2	137.9 (2)
H7A—C7—H7B	107.0	C3—N3—C4	110.4 (3)
O1—C8—C7	114.5 (4)	C3—N3—Cu2	108.1 (2)
O1—C8—H8A	108.6	C4—N3—Cu2	114.4 (2)
C7—C8—H8A	108.6	C3—N3—H3C	107.9
O1—C8—H8B	108.6	C4—N3—H3C	107.9
C7—C8—H8B	108.6	Cu2—N3—H3C	107.9
H8A—C8—H8B	107.6	C4—N4—C7	114.3 (3)
N5^i^—Cu2—N5	180.0	C4—N4—C5	117.2 (3)
N5^i^—Cu2—N3	84.99 (12)	C7—N4—C5	118.8 (3)
N5—Cu2—N3	95.01 (12)	C6—N5—C5	114.6 (3)
N5^i^—Cu2—N3^i^	95.01 (12)	C6—N5—Cu2	107.20 (19)
N5—Cu2—N3^i^	84.99 (12)	C5—N5—Cu2	114.4 (2)
N3—Cu2—N3^i^	180.0	C6—N5—H5C	106.7
N5^i^—Cu2—N1^i^	94.12 (11)	C5—N5—H5C	106.7
N5—Cu2—N1^i^	85.88 (11)	Cu2—N5—H5C	106.7
N3—Cu2—N1^i^	89.19 (10)	C8—O1—H1C	109.3
N3^i^—Cu2—N1^i^	90.81 (10)	H2A—O2—H2B	108.2
N5^i^—Cu2—N1	85.88 (11)	H3D—O3—H3E	109.5
N5—Cu2—N1	94.12 (11)	H4D—O4—H4E	109.5
N3—Cu2—N1	90.81 (10)		
			
N4—C7—C8—O1	−22.2 (6)	N3—C4—N4—C5	−70.7 (4)
N5^i^—Cu2—N1—C1	129.7 (4)	C8—C7—N4—C4	120.0 (4)
N5—Cu2—N1—C1	−50.3 (4)	C8—C7—N4—C5	−94.9 (4)
N3—Cu2—N1—C1	−145.4 (4)	N5—C5—N4—C4	66.8 (4)
N3^i^—Cu2—N1—C1	34.6 (4)	N5—C5—N4—C7	−77.3 (4)
C6^i^—C3—N3—C4	−166.9 (3)	C3^i^—C6—N5—C5	168.3 (3)
C6^i^—C3—N3—Cu2	−41.1 (3)	C3^i^—C6—N5—Cu2	40.2 (3)
N4—C4—N3—C3	−178.4 (3)	N4—C5—N5—C6	−175.7 (3)
N4—C4—N3—Cu2	59.5 (4)	N4—C5—N5—Cu2	−51.3 (4)
N5^i^—Cu2—N3—C3	14.8 (2)	N3—Cu2—N5—C6	165.6 (2)
N5—Cu2—N3—C3	−165.2 (2)	N3^i^—Cu2—N5—C6	−14.4 (2)
N1^i^—Cu2—N3—C3	109.0 (2)	N1^i^—Cu2—N5—C6	−105.5 (2)
N1—Cu2—N3—C3	−71.0 (2)	N1—Cu2—N5—C6	74.5 (2)
N5^i^—Cu2—N3—C4	138.2 (3)	N3—Cu2—N5—C5	37.4 (3)
N5—Cu2—N3—C4	−41.8 (3)	N3^i^—Cu2—N5—C5	−142.6 (3)
N1^i^—Cu2—N3—C4	−127.6 (2)	N1^i^—Cu2—N5—C5	126.3 (3)
N1—Cu2—N3—C4	52.4 (2)	N1—Cu2—N5—C5	−53.7 (3)
N3—C4—N4—C7	75.0 (4)		

Symmetry codes: (i) −*x*+2, −*y*+1, −*z*; (ii) −*x*+2, −*y*+1/2, *z*; (iii) −*y*+5/4, *x*−3/4, −*z*+1/4; (iv) *y*+3/4, −*x*+5/4, −*z*+1/4.

### Hydrogen-bond geometry (Å, °)

**Table:**

*D*—H···*A*	*D*—H	H···*A*	*D*···*A*	*D*—H···*A*
N3—H3C···N2^i^	0.93	2.44	3.261 (4)	147
N5—H5C···O2^v^	0.93	2.38	3.291 (8)	165
O1—H1C···O4^vi^	0.85	2.48	3.149 (7)	136
O4—H4D···N2^vii^	0.85	2.11	2.729 (6)	129
O4—H4E···O2^viii^	0.85	2.47	3.279 (9)	159

Symmetry codes: (i) −*x*+2, −*y*+1, −*z*; (v) *y*+3/4, −*x*+3/4, *z*−1/4; (vi) −*y*+5/4, *x*+1/4, −*z*+5/4; (vii) *x*−1, *y*, *z*+1; (viii) *y*−1/4, −*x*+1/4, −*z*+5/4.
